# The effect of hippocampal NR2B-containing NMDA receptors on chronic cognitive dysfunction in rats with temporal lobe epilepsy

**DOI:** 10.1186/s42494-022-00111-2

**Published:** 2023-01-03

**Authors:** Xiaoqing Luo, Cheng Li, Xiaoli Yu, Guangtao Kuang, Xiaolu Wang, Jufang Liang, Jun Jiang

**Affiliations:** Department of Electrophysiology, Wuhan Children’s Hospital (Wuhan Maternal and Child Healthcare Hospital), Tongji Medical College, Huazhong University of Science and Technology, No.100 of Hong Kong Road, Jiang ’an District, Wuhan, 430016 China

**Keywords:** Hippocampus, NR2B-containing NMDA receptors, Chronic cognitive dysfunction, Learning and memory, Temporal lobe epilepsy

## Abstract

**Background:**

We have previously reported that hippocampal long-term potentiation (LTP) was suppressed in temporal lobe epilepsy (TLE) rats. The N-methyl-D-aspartic acid receptors containing 2B subunit (NR2B-NMDARs) are indispensable to induce the LTP of hippocampus. However, it is still unknown whether the NR2B-NMDARs are implied with the cognitive dysfunction in TLE rats.

**Methods:**

The TLE model was lithium chloride-pilocarpine (li-pilo) model. Morris water maze test was used to evaluate the cognitive function of epileptic rats. Expression of hippocampal NR2B-NMDAs was evaluated by western blotting. Stereotactic injection of NMDA, an agonist of NR2B-NMDARs, into the hippocampus of TLE rats was used to investigate the role of NR2B-NMDARs on cognitive dysfunction.

**Results:**

Cognitive function TLE rats was significantly reduced compared with controls in the Morris water maze test (*P* < 0.05). Western blotting data showed the down-expression of hippocampal NR2B-NMDARs and p-NR2B in TLE rats compared with the control (*P* < 0.05). In addition, hippocampal stereolocalization of NMDA injection improved partially the learning and memory in TLE rats (*P* < 0.05).

**Conclusions:**

Down-expression and low activity of hippocampal NR2B-NMDARs may be implied with chronic cognitive dysfunction in TLE rats.

## Background

Epilepsy is a common chronic neurological disorders with several associated comorbidities [1, 2]. Cognitive dysfunction is one such comorbidity that affects epilepsy prognosis and quality of life [3, 4]. More and more clinical data revealed that cognitive impairment is one of the most common complications of epilepsy. The etiology of epilepsy-related cognitive dysfunction remains unclear, and its pathogenesis needs to be further studied [2, 5, 6]. Temporal lobe epilepsy (TLE) -related cognitive impairment is mainly manifested by deficits in attention, executive function and memory function [7, 8]. Previous studies on the pathogenesis mainly focused on epileptic factors, including age of epilepsy, etiology, type of epilepsy, epileptic syndrome, and duration of epilepsy. In recent years, clinical experts have found that the cognitive impairment of some TLE patients has not been significantly improved, and sometimes even continues to worsen.

It has been reported that hippocampal sclerosis was one of the most common lesion in TLE. The hippocampal long-term potentiation (LTP) has been regarded as the neuronal basis for learning and memory [9, 10]. The N-methyl-D-aspartate receptor (NMDAR), including NR1 subunit and NR2A-D subunits, is a ionotropic glutamate receptor. LTP is an important foundation for learning and memory, and the NR2B-containing NMDARs (NR2B-NMDARs) was important to induce the LTP [10, 11]. We have previously found that the hippocampal LTP is significantly inhibited in TLE rats with chronic cognitive dysfunction [12]. However, whether the NR2B-NMDARs are involved in the formation of cognitive impairment is still unclear in TLE rats.

## Materials and methods

### Animal model of TLE

Animals were randomly assigned into the TLE and control groups on postnatal day 21 (P21) from the Department of Experimental Animal Center, Tongji Medical college, Huazhong University of Science and Technology. Both groups received intraperitoneal injection of 3 mEq/kg lithium chloride and 25 mg/kg pilocarpine. The control group received lithium chloride and saline. Seizures were graded on the Racine scale. Only animals with a Racine score of 3 or higher were used in the following experiments. Status epilepticus (SE) was blocked by chloraldurate (3 ml/kg, i.p.). The animal experimental procedures were all approved by the Animal Care and Use Committee of Huazhong University of Science and Technology (2019013).

### Assessment of chronic cognitive dysfunction

Morris water maze test, mainly composed of a escape platform and a pool with stainless steel wall, was used to evaluate cognitive function of epileptic rats. Rats can find the escape platform 2 cm beneath the water surface by remembering the conspicuous landmarks on the walls. The rat was allowed 120 s to find the escape platform, and 20 s to stay on the platform. An overhead video camera monitored the motion trail of the rats, and the water maze software used to analyse the data.

### Intra-hippocampal injection of NMDA

A stainless steel guide cannula (0.65 mm outer-diameter) was stereotaxically placed to direct into the CA1 region of the hippocampus under anesthesia. According to the Paxinos and Watson Atlas, the target position of the hippocampus is 4.0 mm posterior to the bregma, 2.8 mm lateral to the midline and 2.5 mm above the skull surface [13]. A stainless steel injection tube (0.4 mm diameter) was directly inserted into the guide cannula at 0.5 mm beyond the tip of the latter.

### Western blotting

An equivalent amount of protein (50 g) was isolated from the 2-month-old rat hippocampus and transferred to PVDF membrane by electrophoresis (Invitrogen, Carlsbad, CA). The protein was then overnight combined with mouse anti-NR2B monoclonal antibody (1:500, Abcam, Cambridge Science Park, England), rabbit anti-NR2B (pTyr1472) polyclonal antibody (1:1000, Abcam) or mouse anti-β-actin antibody (1:1000, Abcam). The blots were visualized with peroxidase with peroxidase-conjugated goat anti-mouse IgG (1:5000, Abcam) or goat anti-rabbit IgG (1:5000, Beijing zhongshan Jinqiao Biotechnology Co., Ltd). The band intensity of NR2B and NR2B (pTyr1472) was normalized to the band intensity of β-actin.

### Statistical analysis

All data are were expressed as mean ± SEM and analyzed using the SPSS 10.0 software. One-way repeated measures analysis of variance (ANOVA) was used to assesse cognitive function between control and TLE rats. Unpaired *t*-test was used to analyze western blot results. One- or two-way repeated measures analysis of variance (ANOVA) followed by Bonferroni *post-hoc* test was used for multiple comparisons after NMDA injection in the hippocampus. *P* < 0.05 was considered to be statistically significant.

## Results

### Impaired cognitive dysfunction in TLE rats

**Fig. 1:**
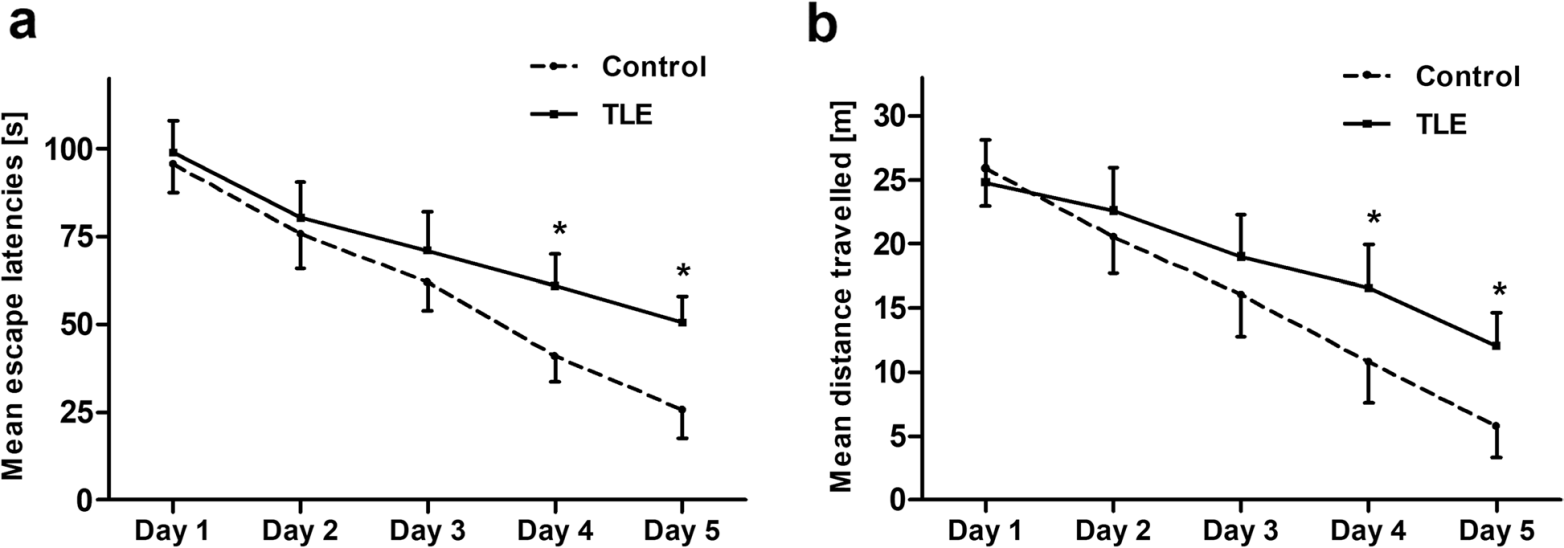
Cognitive dysfunction in rats with temporal lobe epilepsy (TLE). There were significantly higher escape latencies (**a**) and longer distances travelled (**b**) in the TLE rats on days 4 and 5 of training in Morriz water maze. *n*=6 for each group; *P<0.05 *vs* controls

The rats model was induced by injection of li-pilo on P20, and learning and memory ability was estimated on P80. On days 4 and 5 of training in Morris water maze test, TLE rats were significantly different from controls in terms of escape latency and and mean distance travelled (day 4: escape latentcy: TLE 61.14 ± 9.02 s, control 41.01 ± 7.21 s; mean distance travelled: TLE 16.56 ± 3.41 m; controls 10.87 ± 3.17 m; day 5: escape latentcy: TLE 50.54 ± 7.41 s, control 25.74 ± 8.0 s; mean distance travelled: TLE 12.01 ± 2.61 m; controls 5.75 ± 2.47 m; ANOVA followed by unpaired *t*-test, *P* < 0.05, Fig. 1).

### Down-regulation of hippocampal NR2B subunit of TLE rats

**Fig. 2:**
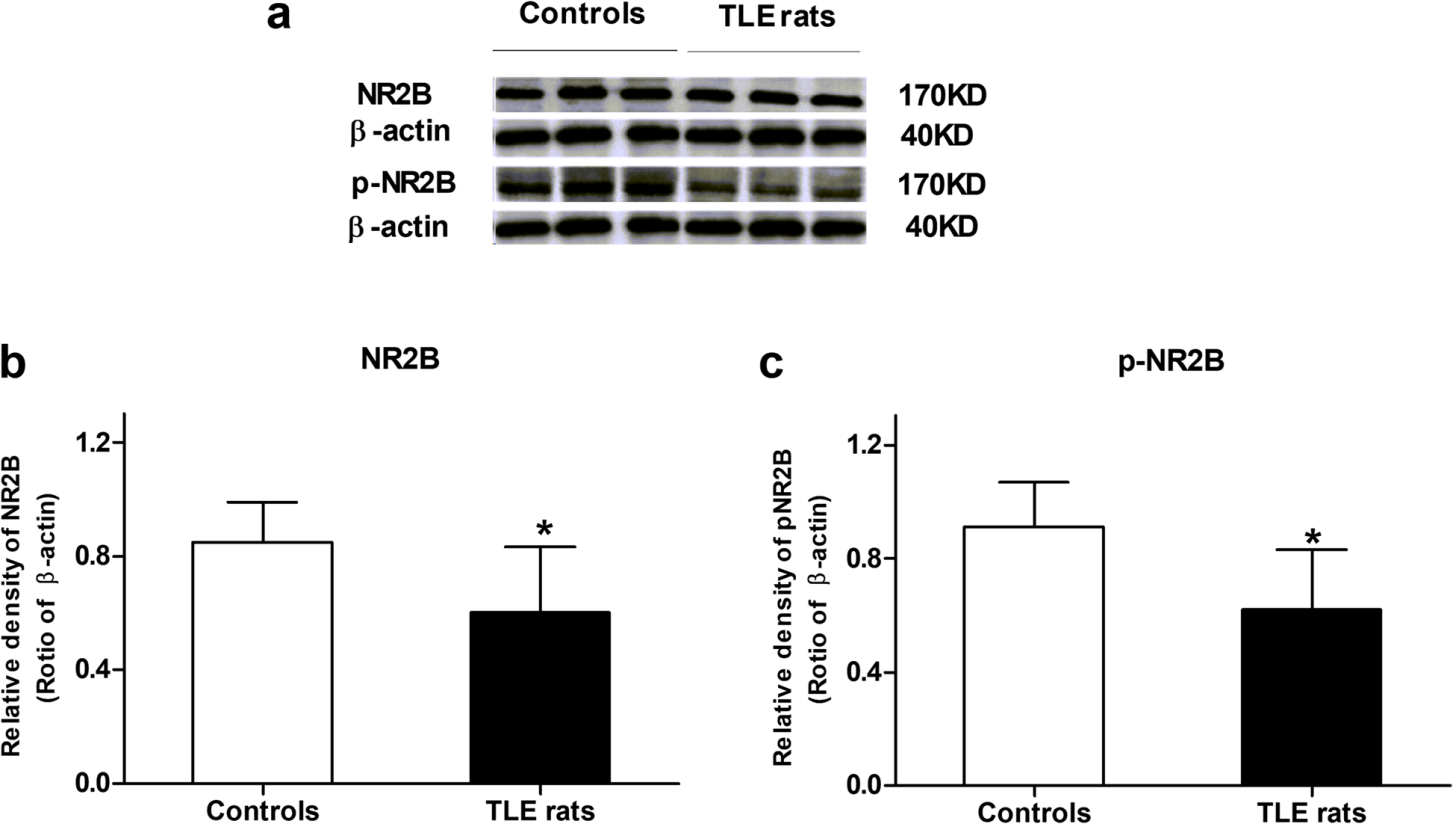
Expression of hippocampal NR2B-containing NMDA receptors in rats. **a** Western blot results showed decreased protein levels of NR2B and p-NR2B in TLE rats (**b** and **c**). Quantification of bands in A. *n* = 3 in each group. *P < 0.05 *vs* control

Western blot results showed that the hippocampal NR2B subunit was significantly decreased by 29.41% in TLE rats compared with the control (0.85 ± 0.14 *vs* 0.60 ± 0.23; unpaired *t*-test, *P* < 0.05, Fig. 2), implying that the down regulation of NR2B subunit may be implicated in cognitive dysfunction in TLE rats. The tyrosine-phosphorylated hippocampal NR2B subunit also decreased by 31.86% in TLE rats as compared with the control (0.91 ± 0.16 *vs* 0.62 ± 0.21; unpaired *t*-test, *P* < 0.05, Fig. 2).These data suggested that the reduced phosphorylation of hippocampal NR2B subunit may play a role in cognitive dysfunction in TLE rats.

### Improvement of learning and memory ability after intervention NMDA treatment in TLE rats

**Fig. 3:**
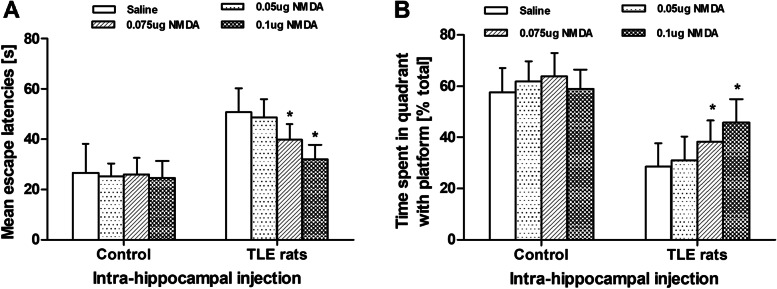
Bilateral intra-hippocampal injection of NMDA improved cognitive function in TLE rats. **a** The mean escape latency was decreased after injection of NMDA in TLE rats in water maze. **b** The time spent in the target quardrant was extended after injection of NMDA in TLE rats in water maze. *n* = 6 for each group; *P < 0.05, *vs* vehicle, Bonferroni *t* test

Bilateral intra-hippocampal injection of NMDA (0.05, 0.075, and 0.1 μg) had no obvious effect on the escape latentcy and the time spent in target quadrant in controls (*P* > 0.05, Fig. 3). For TLE rats, there was no obvious change in the escape latentcy between 0.05 μg NMDA and vehicle treatment (*P* > 0.05, Fig. 3). The escape latentcy in TLE rats was decreased by 21% on day 5 (from 50.74 ± 9.58 to 39.92 ± 6.05) and 37% on day 5 (from 50.74 ± 9.58 to 32.04 ± 5.57), respectively, after injection of 0.075 μg and 0.1 μg NMDA as compared with vehicle (*P* < 0.05, Fig. 3). The time spent in target quadrant in TLE rats was prolonged by 34% on day 5 (28.64% ± 9.17% *vs* 38.32% ± 8.17%) and by 60% on day 5 (from 28.64% ± 9.17% *vs* 45.72% ± 9.15%) after injection of 0.075 μg and 0.1 μg NMDA, as compared with the vehicle (*P* < 0.05, Fig. 3).

## Discussion

In the experiment we studied the the correlation between hippocampal NR2B receptors and cognitive impairment in TLE rats. First, Morris water maze test was used to verify cognitive dysfunction in adult TLE rats. Second, the levels of NR2B protein and tyrosine phosphorylated protein in the hippocampus of TLE rats with cognitive dysfunction were significantly reduced. Third, the application of NMDA can improve cognitive impairment in TLE rats. These data suggest that the decreased phosphorylation of hippocampal NR2B subunits may be implied with the cognitive dysfunction of TLE rats.

More and more clinical evidence has indicated that epilepsy shave a high risk of presenting cognitive impairments such as memory, sustained attention, motor skills, executive function and language [3, 14]. It is widely accepted that approximately 70% of patients with TLE have some degree of cognitive impairment [14]. It was reported that multiple factors may adversely affect cognition in epilepsy, including the underlying etiologies of epilepsy, the seizures themselves, and the antiepileptic drugs [15]. There is growing evidence that uncontrolled seizures can lead to cognitive deterioration [16]. Our experimental data provide a possible molecular mechanism for epilepsy associated with cognitive impairment.

Several experiments in mutant mice have shown that LTP is dependent on the NR2B subunit [17, 18]. Our study revealed the decreased protein expression hippocampal NR2B protein and p-NR2B in TLE rats. There are same and variable results about expression changes of NR2B receptors among these studies. Pratt et al. reported that the NR2B subunit mRNAs were bilaterally reduced in dentate gyrus granule cells from partially kindled rats (10 stimulations) [19]. In another study, mRNA levels of the NR2B subunit in supramasmatic nucleus neuroendocrine cells increased one month after ignition one month after kindling [20]. Mathern et al. reported higher levels of hippocampal NR2B immune response in the self-sustaining marginal state epileptic rats compared with the controls [21]. The differences in results may be related to differences in animal models, brain regions, and NR2B detection time. And the difference could be partly due to the different focus of our research on the pathogenesis of cognitive impairment associated with epilepsy, and more attention has been paid to cognitive impairment than to the number and severity of seizures in the research process. In order to be more rigorous, the frequency and severity of epileptic seizures should be paid as much attention as cognitive impairment in subsequent studies.

NR2B is a subunit of NMDAR2, and NMDA is an agonist of NMDARs. In our study, NMDA dose-dependently improved the cognitive dysfunction in TLE rats. These results supported the notion that low expression and low activity of NR2B-NMDARs may be implied with suppression of hippocampal LTP to lead to the cognitive dysfunction in TLE rats. However, this conclusion that up-regulated NR2B can improve memory basing on the results of NMDA administration cannot be drawn due to lack of detection of NR1 and other subunits of NR2. Since NMDA is a nonspecific agonist of NR2B-NMDARs, more further study is needed to reveal the mechanisms underlying the action of NMDA in cognitive dysfunction in TLE rats.

In this study, NMDA did not completely restore the cognitive function of TLE rats, which may be related to the presence of other signaling molecules in brain associated with cognitive function [22, 23]. Therefore, further studies are required to comprehensively understand the mechanisms of cognitive dysfunction in epilepsy.

## Conclusions

The low expression and low activity of hippocampal NR2B-NMDARs may be implied with chronic cognitive dysfunction in TLE rats.

## Data Availability

The datasets during and/or analysed during the current study available from the corresponding author on reasonable request.

## Abbreviations

IP: Intraperitoneally
li-pilo: Lithium chloride- pilocarpine
LTP: Long-term potentiation
NR2B-NMDAs: N-methyl-D-aspartic acid receptors 2B subunits
PN21: Postnatal day 21
SE: Status epilepticus
TLE: Temporal lobe epilepsy
